# Fibrillin-2, tenascin-C, matrilin-2, and matrilin-4 are strongly expressed in the epithelium of human granular and lattice type I corneal dystrophies

**Published:** 2012-07-18

**Authors:** Eszter Szalai, Szabolcs Felszeghy, Zoltán Hegyi, László Módis, András Berta, Kai Kaarniranta

**Affiliations:** 1 Department of Ophthalmology, University of Debrecen, Medical and Health Science Center, Debrecen, Hungary; 2 Department of Anatomy, Histology and Embryology, University of Debrecen, Medical and Health Science Center, Debrecen, Hungary; 3 Department of Ophthalmology, Institute of Clinical Medicine, University of Eastern Finland, Kuopio, Finland; 4 Department of Ophthalmology, Kuopio University Hospital, Kuopio, Finland

## Abstract

**Purpose:**

To determine the extracellular matrix proteins involved in the formation of human granular and lattice type I corneal stromal dystrophies, the expression patterns of fibrillin-2, tenascin-C, matrilin-2, and matrilin-4 were compared in human corneal stromal dystrophy samples.

**Methods:**

Ten cases of granular dystrophy, 7 cases of lattice dystrophy, and 6 normal corneal buttons collected during corneal transplantation were examined for their expression patterns of fibrillin-2, tenascin-C, matrilin-2, and matrilin-4 by immunohistochemistry.

**Results:**

Highly elevated fibrillin-2, tenascin-C, matrilin-2, and matrilin-4 were observed in the epithelial layer of both granular and lattice type I dystrophies. Fibrillin-2, tenascin-C, and matrilin-4 in the granular dystrophy and all antibodies in the lattice dystrophy showed statistically significant staining in the corneal stroma (p<0.05). Interestingly, fibrillin-2, matrilin-2, and matrilin-4 stained significantly in amyloid plaques of lattice type 1 dystrophy.

**Conclusions:**

Fibrillin-2, tenascin-C, matrilin-2, and matrilin-4 may be markers of the pathogenesis of either granular or lattice type I corneal dystrophy, as revealed by immunohistochemical analysis. Each molecule seems to be involved in the regeneration and reorganization of the corneal matrix in granular and lattice type I dystrophies.

## Introduction

Stromal corneal dystrophies are primary, genetically determined, bilateral, noninflammatory disorders affecting the stromal layer of the cornea. Granular type I corneal dystrophy (corneal dystrophy Groenouw type I, OMIM 121900) is an autosomal-dominant disease characterized by multiple stromal opacities [1]. Its onset usually occurs during childhood, and the rate of clinical disease progression varies between individuals. Lattice type I cornea dystrophy (Biber-Haab-Dimmer dystrophy, OMIM 122200) has autosomal-dominant inheritance and typically presents in the first decade of life [1]. Both granular and lattice type I stromal dystrophies have been linked to allelic mutations in the transforming growth factor-β (TGF-β) induced gene-h3 (*BIGH3*) on chromosome 5q31 [2-4]. The abnormal gene product (protein) accumulates in the corneal layers and forms deposits that are generally characteristic of the corneal dystrophy type [5].

The corneal stroma is composed of keratocytes (2%–3% of the total stromal volume) [6], extracellular matrix (ECM) molecules and stromal nerves. Corneal transparency and function are based on the special composition and assembly of the extracellular matrix structures as well as on the cell-cell and cell-ECM interactions [7]. Fibrillins play an important role in maintaining tissue integrity and homeostasis through the modulation of TGF-β and bone morphogenetic protein signaling [8]. Fibrillin-2 binds to other ECM proteins, forming microfibrils, and is mostly expressed during embryogenesis [9]. Tenascin-C is a hexameric ECM glycoprotein, and its expression is restricted to the fetal period of development [10]. It is responsible for various dynamic cellular activities, including cell adhesion [11], de-adhesion [12,13], inflammation [10], tissue remodeling [10], angiogenesis [14], migration [10], proliferation [15], and growth [11]. Enhanced expression of both fibrillin-2 and tenascin-C has been observed in adults with fibroproliferative conditions, such as wound healing and sclerosis [9,10,16]. Matrilin-2, discovered by Deák et al. [17], is the largest member of an extracellular matrix adaptor protein family of proteins that contain von Willebrand factor A-like domains [18]. Matrilins can connect to different types of collagenous and noncollagenous ECM structures, participate in various protein–protein interactions and, thus, determine the tissue integrity [19]. Matrilin-4 is the most recently identified member of the matrilin superfamily [20]. The trimer matrilin-4 is a component of dense and loose connective tissues, bone, articular cartilage and nervous tissues and associates with basement membranes [21].

To the best of our knowledge, this is the first study to determine the expression pattern of fibrillin-2, tenascin-C, matrilin-2, and matrilin-4 in these corneal stromal dystrophies.

## Methods

### Patients and tissue specimens

Tissue samples were obtained from 12 patients who underwent penetrating keratoplasty. Donor corneas obtained from the Cornea Bank Debrecen (Department of Ophthalmology, University of Debrecen, Medical and Health Science Centre) served as normal control corneas. The study included 23 total cases: 10 cases of granular type I dystrophy (7 patients), 7 cases of lattice type I dystrophy (5 patients), and 6 corneal buttons (6 patients) with a normal fibrillin-2, tenascin-C, matrilin-2, and matrilin-4 staining profile.

### Antibodies

Monoclonal mouse antibodies against fibrillin-2 (clone 48; MAB2642, 1:250; Millipore, Bedford, MA) and tenascin-C (DB7; ab86182, 1:50; Abcam, Cambridge, UK), and polyclonal rabbit antibodies against matrilin-2 (ARP57667_P050, 1:300; Aviva Systems Biology, Corp., San Diego, CA) and matrilin-4 (ab106379, 1:300; Abcam) were used for immunohistochemistry.

### Light microscopy

After corneal button removal, tissue samples were immediately fixed in formalin (10%, pH 7.2) and embedded in paraffin, and then 5−7 µm thick longitudinal sections were cut (Leitz 1208 microtome; Wild Leitz, GmbH, Wetzlar, Germany). Paraffin sections were mounted on Superfrost Plus slides (Menzel-Gläser, Germany) and left to dry overnight. Sections were deparaffinized with xylene and rehydrated through a graded series of ethanol and distilled water. For light microscopy analysis after rehydration, the sections were stained with hematoxylin and eosin (H&E) according to the manufacturer’s protocol and covered with DPX (BDH Laboratory Supplies, Poole, England). Congo Red staining was applied to show the presence of amyloid.

### Analysis of staining

The histological morphology of the corneal samples was evaluated by two independent researchers (E.S., S.F.) at 20× magnification in routine hematoxylin-eosin-stained and in Congo Red sections from each individual cases.

### Immunohistochemical staining

For immunohistochemical analysis after rehydration, slides were treated with 3% hydrogen peroxide to block the endogenous peroxidase activity. For immunostaining, Histostain-Plus kits for mouse and broad-spectrum primary antibodies (Invitrogen, Camarillo, CA) were used. The sections were incubated in serum-blocking solution for 30 min at room temperature. Fibrillin-2, tenascin-C, matrilin-2, and matrilin-4 primary antibodies were applied overnight at dilutions of 1:250, 1:50, 1:300, and 1:300, respectively, in TBS (pH 7.4) at 4 °C. Then, the slices were washed twice with TBS, and the biotinylated secondary antibody (1:200 dilution) was applied for 30 min at room temperature. After washing with TBS, the samples were treated with enzyme conjugate for 30 min at room temperature. Reactions were visualized by the diaminobenzidine (DAB) immunoperoxidase technique using a DAB detection kit (Invitrogen). All kit solutions were diluted in TBS, and the incubations for the DAB chromogen procedure were performed in humid chambers at room temperature according to the manufacturer’s protocol. Nuclei were counterstained with Harris’ hematoxylin (Merck, Darmstadt, Germany). The sections were dehydrated in a graded series of ethanol and xylene and mounted with DPX (BDH Laboratory Supplies). The slides were observed with a light microscope (BX40; Olympus, Tokyo, Japan) equipped with a digital camera (DP50; Olympus), and digitized images were processed using Adobe Photoshop CS4 (Adobe Systems Inc., CA).

We performed preabsorption reaction of matrilin-2 antibody using specific blocking peptide (Aviva Systems Biology, San Diego). The lack of immunostaining in these sections confirms the specificity of our antibody. This type of negative control would be ideal and necessary in the characterization and evaluation of antibodies used but it was not possible to obtain the purified antigen from companies, therefore control sections were stained in same way but the primary antibody was omitted and replaced by non-immune IgG (Sigma Aldrich, St. Louis, MO). No immunostaining was observed in these sections indicating that our immunostaining protocol is specific.

### Immunofluorescence

To examine the colocalization of matrilin-2 and fibrillin-2 in corneal epithelial cell double immunostaining protocol has been performed. Tissue sections were fixed as described above. To prevent the nonspecific staining, before the antibody treatments the slides were incubated in serum-blocking solution for 1 h at room temperature. Subsequently, the slides were incubated with a mixture of antibodies, that contained anti-matrilin-2 (diluted 1:250) and anti fibrillin-2 (diluted 1:300) antibodies. After 2 days long incubation at 4 °C with the primary antibody solution the slides were rinsed in PBS and, the mixture of secondary antibodies (Alexa Fluor 488 anti-mouse Ig and the Alexa Fluor 546 anti-rabbit Ig; Invitrogen, Camarillo, CA) has been placed to samples for 2 h at room temperature. The slides were washed and the nuclei were stained using DAPI (4,6-diamidino-2-fenilindole, dihydrocloride; 1:1,000; Molecular Probes Camarillo, CA) in PBS containing 0.1% saponin for 30 min and covered with Vectashield mounting Media (Vector Laboratories, Burlingame, CA). As negative controls for each double label, the same procedure as above was followed except that one of the primary antibodies was omitted. No immunostaining was seen in these sections [22].

### Confocal microscopy

Acquired and presented images were representative of all the samples examined with a Olympus FV1000 confocal microscope (Olympus, Tokio, Japan). Using 60× oil immersion lens single 1 µm thick section were scanned. The confocal settings were identical for all scans and kept constant during imaging. The recorded images were processed by Adobe Photoshop CS4 software.

### Computer-assisted image analysis

The immunostained results were recorded as digital images using the setup described above. From each immunohistochemical slide, representative fields (100× objective) were captured. The settings of the microscope and camera were kept constant during capturing of the pictures, and digitalized images were analyzed in Image J software with macroinstructions for analyzing each captured area. The total intensity of DAB developed immunohistochemical staining of different antibodies used (Fibrillin-2/Tenascin-C/Matrilin-2 and-4), was measured for each region of interest (ROI). Pictures were recorded from 5 different randomly selected individual cases in each groups and on 5 sections from each of them independent gray intensity of ROIs (size approximately 5 μm^2^) was determined at two different locations of the corneal epithelium (basal and suprabasal cell layers) and in the subepithelial and deep zones of the corneal stroma.

### Statistical analysis

Data were analyzed using the SPSS software version 11.0.05 (SPSS Inc., Chicago, IL). Significant differences in immunohistochemical staining intensity were determined by the Mann–Whitney U-test; differences with p<0.05 were considered significant.

## Results

### Histopathology

**Figure 1:**
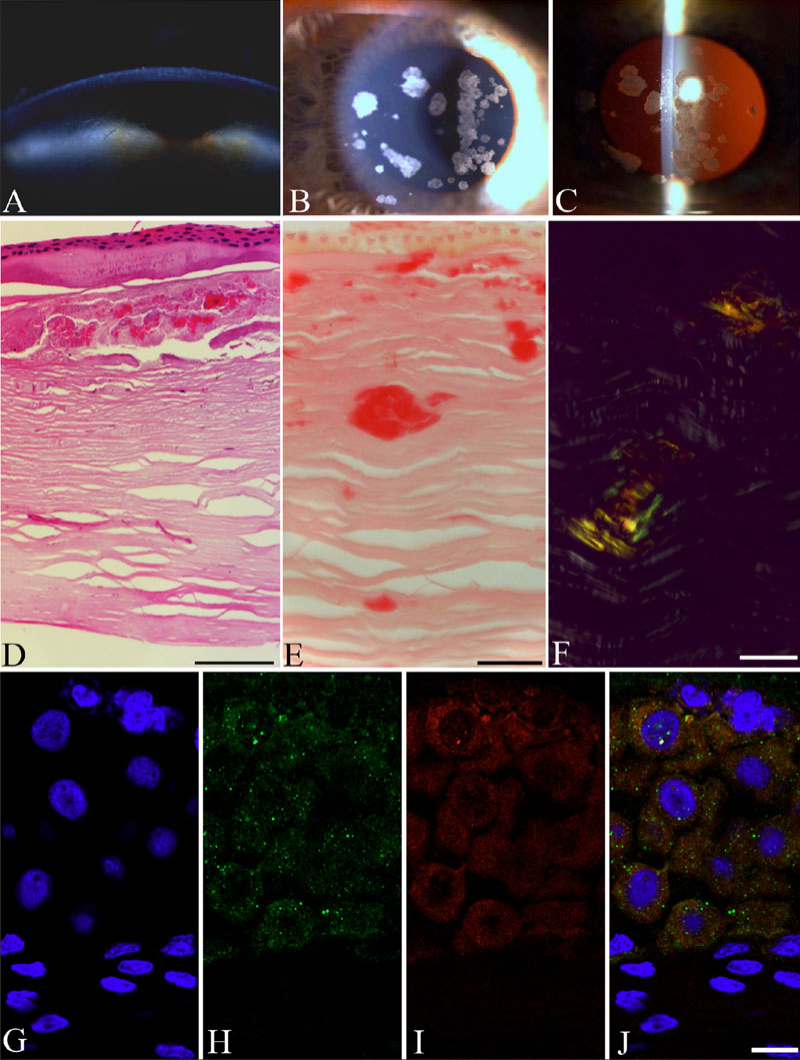
Granular and lattice type I dystrophies: biomicroscopic and histopathologic findings. The clinical appearance of lattice corneal dystrophy (**A**). The branching dots and lines, representing amyloid deposits, are typical in the corneal stroma. Granular corneal dystrophy under slit lamp and retroillumination (**B**). Well defined, white granules are present in the central corneal stroma (**C**). Histological section of the granular type I corneal button (H&E) (**D**). The deposits are observed in the sub-Bowman layer in the stroma. The section of lattice corneal dystrophy stains weak with Congo Red (**E**). Stromal fusiform amyloids stain with Congo red (**F**) and show birefringence under polarized light. The colocalization of matrilin-2 and fibrillin-2 in normal corneal epithelium is presented in **G**-**J**. High magnification microphotos of single laser scanning confocal optical sections (1 μm) demonstrating the immunolocalization of the anti-matrilin-2 (**H**, green) and anti-fibrillin-2 (**I**, red) antibody in corneal epithelial layer. The nuclei are blue on each images presented (**G**-**J**). Note that no nuclear staining is present on the merged image (**J**) preclude the possibility of nonspecific reaction (internal control). Around the smaller DAPI labeled nucleus yellow color may indicate the colocalization of these two antibodies used. Scale bars for light and polarized microscopic images are 100 μm, for confocal images; 10 μm.

Figure 1 shows the clinical appearance of lattice type I (Figure 1A) and granular type I corneal dystrophy (Figure 1B,C). Figure 1D shows longitudinally sectioned granular type I stromal dystrophy after H&E staining. Typical of granular type I stromal dystrophy, multiple eosinophilic granules (asterisks) in the ground substance of the corneal stroma were observed. Figure 1E shows the amyloid plaques characteristic of lattice type I dystrophy. The deposits were variable in size, located in the mid- to deep stroma. Figure 1F shows the optical anisotropy of amyloids. Note the characteristic yellow-green birefringence after Congo Red staining under polarized light microscopy (arrowheads), indicating that these structures are amyloid in nature. The immunostainings of the anti-matrilin-2 and anti-fibrillin-2 antibodies were confirmed to localize in corneal epithelial layer analyzed by a single laser scanning confocal microscopy (Figure 1G-J).

### Immunohistochemistry

The immunolocalization and staining intensity of fibrillin-2, tenascin-C, matrilin-2, and matrilin-4 in normal, granular type I and lattice type I stromal dystrophy corneas are summarized in Table 1.

**Table 1:** Immunolocalization of fibrillin-2, matrilin-2, matrilin-4, and tenascin-C in normal and stromal dystrophy corneas (maximum and minimum gray levels are 0 and 255, respectively).

	**Control**				**Granular type I dystrophy**				**Lattice type I dystrophy**			
	**Epithelium**		**Stroma**		**Epithelium**		**Stroma**		**Epithelium**		**Stroma**	
**Antibody**	**Suprabasal**	**Basal**	**Subepithelial**	**Deep**	**Suprabasal**	**Basal**	**Subepithelial**	**Deep**	**Suprabasal**	**Basal**	**Subepithelial**	**Deep**
Fibrillin-2	205.97±3.95	188.42±3.06	168.09±5.83	167.66±9.54	116.37±6.34	54.55±2.5	110.30±5.21	122.36±6.83	104.08±4.04	44.78±2.22	158.6±5.37	158.10±6.85
p*					<0.0001	<0.0001	<0.0001	<0.0001	<0.0001	<0.0001	0.001	0.012
Tenascin-C	165.49±5.50	140.41±5.29	163.9±5.54	223.25±3.79	110.12±5.68	49.39±2.27	100.58±5.75	100.69±5.11	150.93±4.62	102.91±5.3	163.34±7.47	156.8±6.19
p*					<0.0001	<0.0001	<0.0001	<0.0001	<0.0001	<0.0001	0.912	<0.0001
Matrilin-2	149.87±4.75	158.29±7.86	130.68±4.49	151.45±6.06	105.5±4.56	62.74±3.41	135.39±6.65	156.41±7.41	131.06±3.5	94.95±4.07	139.01±5.63	140.39±4.55
P*					<0.0001	<0.0001	0.248	0.190	<0.0001	<0.0001	0.002	0.0001
Matrilin-4	162.21±6.55	165.07±7.13	223.09±2.41	222.54±2.84	102.54±4.82	85.13±3.82	119.48±4.76	115.56±4.59	125.90±4.2	91.26±4.46	134.44±5.93	132.28±4.13
P*					<0.0001	<0.0001	<0.0001	<0.0001	<0.0001	<0.0001	<0.0001	<0.0001

*Mann–Whitney U test (control versus granular/lattice dystrophy).

#### Fibrillin-2

**Figure 2:**
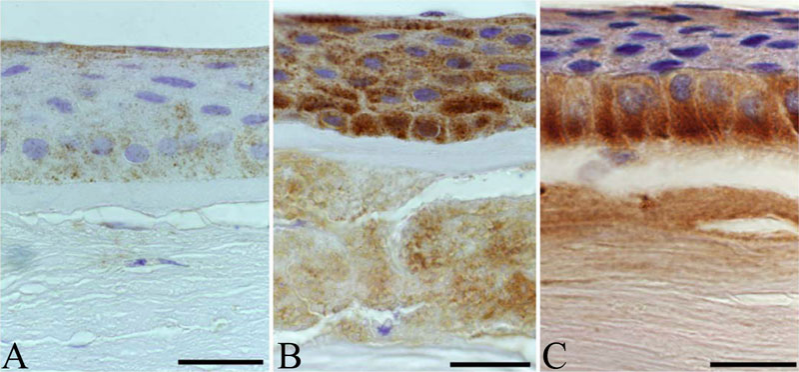
Comparison of expression patterns of fibrillin-2 in healthy, granular type I and lattice type I stromal dystrophy corneal buttons, and the colocalization of matrilin-2 and fibrillin-2 in corneal epithelium. Healthy samples showed mild immunolocalization in superficial and basal epithelial layers (**A**). In addition to the strong epithelial immunopositivity of fibrillin-2 in granular dystrophy, marked labeling was observed in stromal granules (**B**). In lattice corneal dystrophy, continuous cytoplasmic staining was found in the base of the basal columnar cells (**C**). Scale bar 100 μm.

In normal corneal specimens, some corneal epithelial cells showed moderate cytoplasmic immunoreactivity (Figure 2A), although suprabasal and basal epithelial expression was significantly weaker in normal sections than in granular type I dystrophy (p<0.0001 for both; Mann–Whitney U test) and in lattice type I corneal dystrophy (p<0.0001 for both). The expression of fibrillin-2 in the basal cell layer and the superficial squamous cell layer of the corneal epithelium was intense in granular type I dystrophy (Figure 2B). No immunoreactivity was observed in Bowman’s membrane. Stromal deposits showed intense immunolocalization of fibrillin-2, but the ground substance of the stroma between the granules did not stain with the fibrillin-2 antibody (p<0.0001 for both). Nearly as intense cytoplasmic immunoreactivity was found in the basal epithelial cells in lattice type I dystrophy as in those of granular type I dystrophy. Immunopositive cells formed a relatively continuous border between the epithelium and Bowman’s layer (Figure 2C). The anterior stroma and the amyloid plaques showed definitive immunoreactivity for fibrillin-2.

#### Tenascin-C

**Figure 3:**
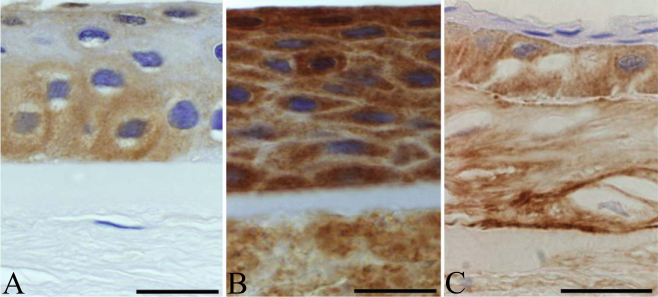
Comparison of the expression patterns of tenascin-C in healthy, granular type I, and lattice type I stromal dystrophy corneal buttons. Cytoplasmic immunolabeling in normal sections was only observed in some basal epithelial cells (**A**). In granular dystrophy, diffuse, marked immunostaining was recorded in the epithelial layers and in the stromal granules (**B**). Basal epithelium showed moderate immunostaining for tenascin-C in lattice dystrophy (**C**). Scale bar 100 μm.

Normal corneal buttons showed mild immunolocalization for tenascin-C in the corneal epithelium (Figure 3A). In granular type I corneal dystrophy, epithelial cells stained intensely for tenascin-C (p<0.0001 for both; Figure 3B). Bowman’s membrane did not display any immunopositivity for this protein. Stromal staining was stronger and statistically significant in the stromal deposits (p<0.0001 for both) compared with between the granules. In basal epithelial cells, tenascin-C showed moderate immunolocalization in lattice type I dystrophy (p<0.0001; Figure 3C). In Bowman’s layer, immunoreactivity was absent. Some mild immunostaining was detectable in the corneal stroma, but the immunoreactivity found in the amyloid deposits was absent or mild and, thus, nonspecific (p=0.912, subepithelial).

#### Matrilin-2

**Figure 4:**
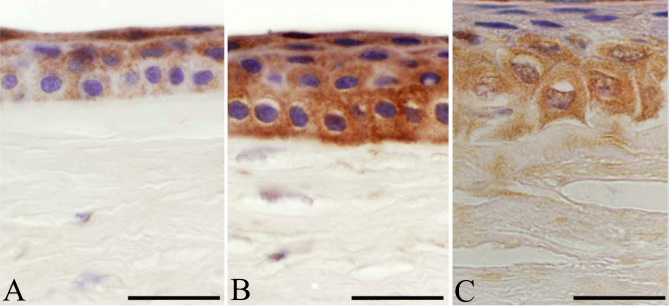
Comparison of the expression patterns of matrilin-2 in healthy, granular type I, and lattice type I stromal dystrophy corneal buttons. In normal controls, matrilin-2 was present in most corneal epithelial layers (**A**). Intense epithelial immunopositivity was observed in granular dystrophy. Focal thinning or loss of Bowman’s layer was also apparent (**B**). Enlarged immunopositive basal cells in corneal epithelium with mild stromal staining were observed in lattice dystrophy (**C**). Scale bar 100 μm.

Mild immunopositivity for matrilin-2 was found in normal samples (Figure 4A). In granular type I dystrophy, corneal epithelium showed intense, statistically significant intracellular reactions for matrilin-2, especially in wings and basal cells (p<0.0001 for both). Figure 4B shows the focal thinning of Bowman’s layer in granular dystrophy. Stromal expression of matrilin-2 was observed, mainly in the corneal granules (p=0.248, subepithelial; p=0.190, deep). In lattice type I corneal dystrophy cases, diffuse but intense immunoreactivity for matrilin-2 was found in the enlarged basal epithelial cells (p<0.0001; Figure 4C). The thinned or absent Bowman’s membrane showed no immunostaining. Corneal stroma in the amyloid deposits was mildly positive, and posterior stroma showed mild to moderate immunoreactivity in some lattice type I dystrophy cases, this difference was statistically significant (p=0.0001).

#### Matrilin-4

**Figure 5:**
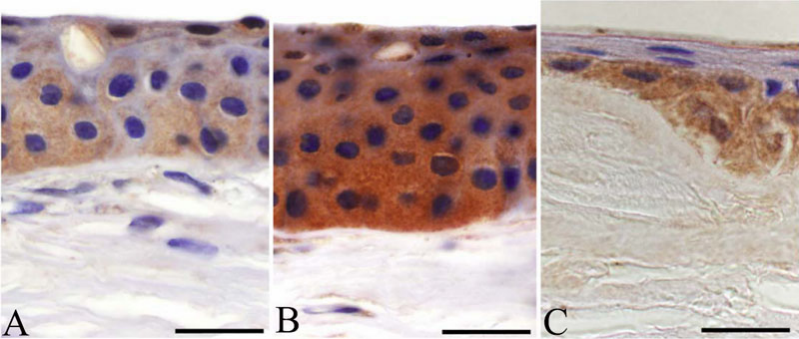
Comparison of the expression patterns of matrilin-4 in healthy, granular type I, and lattice type I stromal dystrophy corneal buttons. Some immunolabeling was recorded in healthy corneal epithelium (**A**). In granular dystrophy, all epithelial layers were positive for matrilin-4, and no Bowman’s layer was present under the epithelial basement membrane (**B**). Altered epithelial cell morphology in the basal layer was observed, with strong basal immunolabeling for matrilin-4 in lattice type I dystrophy (**C**). Scale bar 100 μm.

In normal corneal specimens, mild immunoreactivity was detected for matrilin-4 in corneal epithelium (Figure 5A). In the epithelial layer of granular type I dystrophy, diffuse, intense but discontinuous cytoplasmic immunoreactivity for matrilin-4 was found (p<0.0001 for both). Note the absence of Bowman’s membrane in Figure 5B. Deposits in the corneal stroma were positive for matrilin-4, and some stromal cells between the granules showed an irregular and mild expression pattern (p<0.0001 for both). The superficial and basal epithelial cells displayed statistically significant, mild to moderate immunostaining for matrilin-4 in lattice type I dystrophy cases (p<0.0001 for both; Figure 5C). Bowman’s membrane, where present, was negative for matrilin-4. The anterior and posterior third of corneal stroma (p=0.945 and 0.101, respectively) and amyloid plaques showed intense and statistically significant immunolocalization of matrilin-4 in lattice type 1 dystrophy.

## Discussion

The present study investigated the expression patterns of fibrillin-2, tenascin-C, matrilin-2, and matrilin-4 in granular and lattice type I corneal stromal dystrophy samples. Unexpectedly, significantly higher immunoreactivity was detected for every antibody in the corneal epithelium in both types of dystrophy compared to the normal control samples. In the corneal stroma, stronger immunostaining for tenascin-C was found in the granular type I dystrophy group than in the control samples or lattice dystrophy group.

In granular type I corneal dystrophy, eosinophilic deposits primarily accumulate in the corneal stroma as a result of genetic mutation but also can be found in the epithelial layer [23]. Accordingly, we observed moderate immunopositivity for fibrillin-2 in the basal and superficial epithelial cells. Fibrillins are produced by fibroblasts physiologically in developing or in regenerating tissues. In granular dystrophy, enhanced expression of fibrillin-2 can be explained by the increased ECM production and the multiple functions of fibrillins in ECM stabilization, in which they form microfibrils that associate with basement membranes and elastic fibers [24]. Additionally, modified BIGH3 proteins are thought to promote the adhesion, migration, and proliferation of epithelial cells and fibroblasts more efficiently than the wild type [25-27]. Mutant BIGH3 protein–related peptides contribute to amyloid formation by assembling β-sheet-rich oligomeric structures in lattice type I dystrophy corneas [28]. In accord with other studies, we also demonstrated the appearance of fibrillin-2 in amyloid plaques [29,30]. Basal cells of the corneal epithelium, which secrete the epithelial basement membrane, strongly expressed fibrillin-2 in lattice type I dystrophy, suggesting an ongoing healing process or fibrogenesis. In normal specimens, slight immunolocalization for fibrillin-2 was occasionally observed in some epithelial cells, which was significantly weaker than those in the dystrophy samples. In addition, two papers have reported immunopositivity for fibrillin-2 in the epithelial basement membrane of normal buttons [29,31].

Epithelial erosions have been described both in granular and in lattice type I dystrophy [32]. Increased expression of fibrillin-2 in the corneal epithelial cells may indicate epithelial wound healing. Brinckman et al. [16] attributed the co-expression of fibrillin-2 and tenascin-C in wound healing to altered cell adhesion in ECM. In our study, similar expression patterns of fibrillin-2 and tenascin-C were found in granular type I dystrophy but not in lattice dystrophy. We detected stronger immunolocalization for tenascin-C in corneal epithelial cells and stromal granules in granular type I dystrophy specimens compared to the normal samples. This finding supports our assumption of decreased cell adhesion as a prominent feature of granular type I stromal dystrophy corneas. Tenascin-C immunopositivity in inflammation [33], active scarring [33,34], angiogenesis [35] and bullous keratopathy [36,37] have been documented. In developmental studies, tenascin-C is consistently detected in neonatal corneas, but it is limited to the limbus in adults [38,39]. In lattice type I dystrophy, moderate immunolocalization for tenascin-C was observed in all epithelial layers. One conspicuous finding of this study was the absent or weak immunopositivity of tenascin-C in the amyloid deposits. Similarly to other authors, we did not find this glycoprotein in normal central corneas [31,33,40], although some immunopositivity was observed in the epithelial cell layers. It should be noted that we used the monoclonal antibody DB7 against tenascin-C [41] and that the tenascin-C variants have different expression patterns [33,38].

Matrilins are recently identified adaptor proteins, but few studies have investigated their presence and function in the human cornea [31,39]. Matrilin-2, the non-collagenous basement membrane component, colocalizes with microfibrils and connects to fibrillin-2 [42]. In this study, mild to moderate matrilin-2 expression was observed in the epithelial layer and in the stromal deposits in granular type I dystrophy, the expression patterns of fibrillin-2 and matrilin-2 were similar. This result also supports the notion that the regeneration and reorganization of the ECM that occur in granular type I dystrophy might be analogous to those of the wound healing process. Matrilin-2 functions in early cell differentiation during embryonal development, promotes axonal growth and Schwann cell migration during peripheral nerve regeneration [43], modulates wound healing in the skin [44] and plays a role in tumor development [45,46].

We observed diffuse, marked immunoreactivity for matrilin-2 in the epithelial cells of lattice type I dystrophy samples, but only slight reactivity in the stromal amyloid deposits. Posterior stroma also showed some mild immunostaining for matrilin-2 in lattice stromal dystrophy. Normal corneal epithelium and stroma exhibited mild immunopositivity for matrilin-2; however, two previous studies demonstrated immunolocalization of this oligomeric protein in the epithelial basement membrane in adult corneas [31,39]. Matrilin-4 staining in the stromal granules was less pronounced in granular type I dystrophy than the matrilin-2 reactivity. In contrast, amyloid deposits showed stronger immunolocalization for matrilin-4 than for matrilin-2 in lattice type I dystrophy, highlighting the difference in expression patterns between these two adaptor proteins in this disease. However, matrilin-2 and −4 arise from a common precursor, which suggests a close association between their tissue localization and functional roles [21]. Matrilin-4 is expressed in most tissue locations where any of the other matrilins are present [21]. Further studies focusing on the difference between the roles of matrilin-2 and matrilin-4 are necessary to explain their different expression patterns in granular and lattice type I dystrophies. Matrilin-4 immunoreactivity has been observed in the epithelial basement membrane in adult corneas [31,39], although we only found weak immunostaining in the epithelial cells in control specimens.

In conclusion, fibrillin-2 and tenascin-C are largely restricted to developing fetal tissues but become expressed again in granular type I corneal dystrophy and in lattice type I dystrophy. Fibrillin-2, tenascin-C, matrilin-2, and matrilin-4 may be characteristic of the pathogenesis of either the granular or lattice type I corneal dystrophy, as revealed by immunohistochemical analysis. Each molecule seems to be involved to some degree in the regeneration and reorganization of the corneal matrix in granular and lattice type I dystrophies. However, we observed substantial differences in ECM reconstruction and remodeling between the two dystrophy types. Further experiments are needed to confirm the role of these proteins in the matrix regeneration and repair in corneal stromal dystrophies and to reveal their exact mechanism of action.

## 
